# In Silico Identification of Cholesterol Binding Motifs in the Chemokine Receptor CCR3

**DOI:** 10.3390/membranes11080570

**Published:** 2021-07-28

**Authors:** Evan van Aalst, Jotham Koneri, Benjamin J. Wylie

**Affiliations:** Department of Chemistry and Biochemistry, Texas Tech University, Lubbock, TX 79423, USA; Evan.van-Aalst@ttu.edu (E.v.A.); Jotham.Koneri@ttu.edu (J.K.)

**Keywords:** GPCR, CCR3, chemokine receptor, cholesterol, CRAC motif, CARC motif, Molecular Dynamics, Pylipid

## Abstract

CC motif chemokine receptor 3 (CCR3) is a Class A G protein-coupled receptor (GPCR) mainly responsible for the cellular trafficking of eosinophils. As such, it plays key roles in inflammatory conditions, such as asthma and arthritis, and the metastasis of many deadly forms of cancer. However, little is known about how CCR3 functionally interacts with its bilayer environment. Here, we investigate cholesterol binding sites in silico through Coarse-Grained Molecular Dynamics (MD) and Pylipid analysis using an extensively validated homology model based on the crystal structure of CCR5. These simulations identified several cholesterol binding sites containing Cholesterol Recognition/Interaction Amino Acid Consensus motif (CRAC) and its inversion CARC motifs in CCR3. One such site, a CARC site in TM1, in conjunction with aliphatic residues in TM7, emerged as a candidate for future investigation based on the cholesterol residency time within the binding pocket. This site forms the core of a cholesterol binding site previously observed in computational studies of CCR2 and CCR5. Most importantly, these cholesterol binding sites are conserved in other chemokine receptors and may provide clues to cholesterol regulation mechanisms in this subfamily of Class A GPCRs.

## 1. Introduction

G protein-coupled receptors (GPCRs) are integral membrane proteins comprising seven transmembrane alpha helices (TM), three extracellular (EC), and three intracellular (IC) loops involved in ligand-binding and G protein docking [1]. Chemokine receptors are a subfamily of Class A GCPRs expressed by immune cells that induce chemotaxis of the expressing cell through the binding of small protein agonists to the receptor’s orthosteric binding pocket [2]. Chemokines contain structural disulfide bond motifs formed by N terminal cysteines that dictate subfamily: CC motif, CXC motif, CX_3_C motif, and XC motif [3]. The cognate receptor is therefore defined based on the native ligand(s). Of the chemokine receptors, CCR5 and CXCR4 are best understood given their known critical role in cellular human immunodeficiency virus (HIV) entry [4,5]. Cholesterol binding and dynamic dimerization sites were observed in both receptors from MD simulations [6].

Lipid modulation of GPCR function is well documented and includes anionic phospholipids such as phosphatidyl serine (PS) [7] and phosphatidylinositol-4,5-bisphosphate (PIP_2_) [8] as well as cholesterol [9]. Cholesterol is an allosteric modulator of function and the dynamic glue for GPCR dimerization [9,10,11,12,13]. However, in vitro molecular investigation of dynamic lipid–receptor interactions is quite difficult as techniques such as NMR are limited by heterologous protein yield and stability. Thus, molecular details are largely derived from cholesterol co-crystallization in GPCR crystal structures, which, while informative, do not encode dynamic information. A GPCR-specific Cholesterol Consensus Motif (CCM) was identified in β_2_AR [6] and is conserved in 21% of Class A GCPRs but absent from chemokine receptors [12].

The Cholesterol Recognition/Interaction Amino Acid Consensus (CRAC) motif is a linear cholesterol binding motif characterized as L/V–X_1–5_–Y–X_1–5_–R/K, first identified in the peripheral-type benzodiazepine receptor [14,15]. The reverse sequence, deemed the CARC motif, was later uncovered in the nicotinic acetylcholine receptor and found to have a sequence of R/K–X_1–5_–Y/F–X_1–5_–L/V [15,16]. These motifs are found in a wide range of membrane proteins [17], including membrane scaffold proteins [18], HIV membrane fusion proteins [19], and GPCRs [20]. CRAC/CARC motifs provide the ability to define binding sites into easily translatable motifs, but the applicability of such endeavors in GPCRs is still debated [21]. Indeed, cholesterol is known to crystallize bound to GPCRs lacking a CCM, CRAC, or CARC motif [22,23] and does not always occupy such motifs even when present [24,25]. In chemokine receptors, cholesterol crystalized with CCR9 localized on TM7 [26], but did not appear to bind to the crystal structure of CCR5 [27] or CXCR4 [28], despite inclusion in the lipidic cubic phase. More recently, the Cryo-EM structure of CXCR2 was solved, where cholesterol was located in a pocket formed by TMs 2, 3, and 4 [29]. Computationally, interactions are also typically evaluated on a case-by-case basis [9,10,11,13]. The degree of conservation of potential cholesterol binding motifs within the chemokine receptor subfamily is therefore unknown, and classification may therefore be a worthwhile endeavor.

Molecular Dynamics (MD) simulations are a useful tool for studying such GPCR–lipid interactions. Coarse-grained (CG) MD facilitates the analysis of dynamic interactions on timescales that are typically too computationally demanding to perform on atomistic systems. Such approaches have been implemented to good effect in defining lipid–protein binding sites [30,31,32,33,34,35]. Computation approaches are not, however, limited to defining the locus of lipid–protein interactions. Computation-based drug design makes use of docking suites such as AutoDock [36] and HADDOCK [37] to identify ligand–receptor interactions using experimentally determined structures. Positive hits can be further characterized via MD [38]. So-called rational drug design [39] remains a promising methodology for the development of GPCR-targeting pharmaceuticals. Such computational approaches comprise a critical role in pursuit of this goal.

CCR3 is a chemokine receptor expressed by eosinophils and other immune cells. Binding of its peptide agonists CCL11, 24, 26, and others induces chemotaxis of the expressing cell [40]. CCR3 therefore plays major roles in asthma [41], rheumatoid arthritis [42], and eosinophilic esophagitis [43] as well as cancer metastasis [44,45,46,47]. However, there is no known structure of CCR3, and little is known about how the bilayer environment influences CCR3 function. In this vein, we leverage µs timescale CGMD simulations in the Groningen Machine for Chemical Simulations (GROMACS) [48] using the Martini2.2 forcefield [49,50,51] to investigate CCR3 behavior and plasticity in environments of increasing cholesterol content. We then use Pylipid [30,31,52] analysis of GROMACS simulations to identify lipid–protein contact sites to identify potential cholesterol binding sites in the transmembrane region. Six binding sites were identified with greater than 1 µs cholesterol residency time at 30% *w/w* cholesterol, containing 9 of the 12 CRAC and CARC sites found within CCR3. Using residency time as a criterion, the binding site in the EC clefts of helices 1 and 7 emerged as a site of interest, which will be investigated in future work. Lastly, many of the CRAC/CARC sites identified herein are strongly conserved in other CC and CXC motif chemokine receptors and could therefore serve as a starting place for in vitro and in silico investigation of other systems.

## 2. Materials and Methods

### 2.1. CCR3 Homology Modeling and Structure Validation

The model used in this work was previously presented by van Aalst and Wylie [53]. Here, we present the methodology for the generation and validation of this model in much greater detail. We submitted the full-length CCR3 sequence (Uniprot ID: P51677) to the Baker lab Robetta comparative modeling server [54] using the CCR5 crystal structure PDB 4MBS [27] as the template (Uniprot ID P51681). This template was selected due to the sequence similarity to CCR3 of 54%, 93% sequence coverage, as well as a Global Model Quality Estimate of 0.71 as reported by the Swiss-Model template search [55]. One hundred models were generated and the top 5 based on internal scoring were displayed by Robetta. These models were manually truncated to residues 23–317, leaving helix 8 but removing error-prone regions that were likely to make modeling difficult (Figure S1). We assessed the overall quality of truncated models using the MolProbity scoring system, compared to the template used for modeling (Table S1) [56]. Ramachandran analysis was performed using MolProbity and PROCHECK [57], and Z-score comparison to experimentally determined structures was performed with ProSa [58,59] for both the selected and truncated model and the CCR5 template (Table S1, Figure S2). Small molecules UCB35625 (ZINC13862637) [60], YM344031 (ZINC209227900) [61], SB328437 (ZINC59618358) [62], CH0076989 (ZINC1000177021) [60], Melatonin (ZINC57060), Serotonin (ZINC57058), L-Dopa (ZINC895199), Phenylethylamine (ZINC6579654), Dopamine (ZINC33882), Glutamine (ZINC1532526), Histamine (ZINC388081), and Glycine (ZINC465855) were downloaded from the ZINC database (accessed 1 October 2020) [63] (Figure S3) and, along with the CCR3 model, prepared for docking analysis with AutoDock tools from the MGL Tools package (version 1.5.6, Molecular Graphics Lab, Scripps Research Institute, La Jolla, CA, USA) [64]. For CCR3, water was deleted, polar-only hydrogens were added, and Kollman charges were added. For ligands, the ZINC database file was converted to a pdbqt file with no further processing. Docking was performed with AutoDock Vina [36] using an energy range of 4 and an exhaustiveness of 8. The grid box search space (Table S2) was defined by the identified small molecule agonist/antagonist pocket Y41^1.39^, Y113^3.32^, E287^7.39^ [60], treated as rigid residues.

### 2.2. Coarse-Grained Martini Simulations

The CCR3 model was inserted into membranes consisting of 1-pamlitoyl-2-oleoyl-phosphatidylcholine (PC) only, 5%, 10%, 15%, 20%, and 30% cholesterol (*w/w*) using the CHARMM-GUI [65,66] Martini maker [67,68]. Bilayers were built into rectangular boxes of size 200 nm at 310.15 K and 1 bar by aligning the CCR3 model along the principal z axis, followed by the addition of 150 mM NaCl with molecular replacement and solvation. For each membrane composition, 5 replicate systems [69] were built de novo using identical parameters. Each replicate was individually energy-minimized and equilibrated before production in the GROMACS (version 2020, GROMACS development teams at the Royal Institute of Technology and Uppsala University, Sweden) [48] using the default settings produced by CHARMM-GUI with the Martini2.2 forcefield [49,50,51] (University of Groningen, Groningen, Netherlands). Briefly, minimization and equilibration were performed using the Berendensen Barostat [70], v-rescale thermostat, and the reaction-field method to manage electrostatics [71]. Production was carried out with the same parameters except with the Parrinello-Rahman Barostat [72] for 10 µs per replicate using the Martini2.2 forcefield [49,50,51]. Molecular dynamics parameter (mdp) files for all minimization and equilibration steps were the standard outputs from CHARMM-GUI. Production mdp files were altered solely to change the number of steps to influence the simulation length. Computational time was provided by NMRbox [73] and the Texas Tech University High Performance Computing Center (HPCC).

### 2.3. Receptor–Lipid Analysis

Root Mean Square Deviation (RMSD), Radial Distribution Function (RDF), and Root Mean Square Fluctuation (RMSF) calculations were performed with the gmx rms, gmx rdf, and gmx rmsf functions in GROMACS, respectively, using the default normalization parameters. Receptor RMSD is output as a function of simulation time per replicate. Replicates were averaged together for presentation. RDF calculations were performed for each lipid species in each replicate with respect to the receptor. Data per replicate were averaged for presentation. RMSF values per residue for each replicate were averaged to generate raw RMSF per residue. The residue average in the 0% simulation was subtracted from the average RMSF per residue of each cholesterol-containing simulation to calculate ΔRMSF. Lipid–protein contacts and binding site sorting were analyzed using Pylipid (https://github.com/wlsong/PyLipID; accessed on 1 October 2020, Song, et al. University of Oxford, Oxford, UK,) [30,31,52] with interaction cutoffs of 0.5 and 1.0 nm, start and end, respectively, for both cholesterol and PC.

### 2.4. Receptor Sequence Analysis

CRAC and CARC motif [17] sequences were identified in CCR3 using EMBOSS: fuzzpro (European Bioinformatics Institute, Wellcome Genome Campus, Hixton, Cambridgeshire, UK, accessed on 1 February 2021) and mapped onto snake plots using the GPCR Database (accessed on 1 February 2021) for visualization [74]. Amino acid sequences for each CC and CXC chemokine receptor were accessed from the UniProt database (accessed on 1 February 2021) with the following accession numbers: P32246 (CCR1), P41597 (CCR2), P51677 (CCR3), P51679 (CCR4), P51681 (CCR5), P51684 (CCR6), P32248 (CCR7), P51685 (CCR8), P51686 (CCR9), P46092 (CCR10), CXCR1 (P25024), P25025 (CXCR2), P49682 (CXCR3), P61073 (CXCR4), P32302 (CXCR5), and O00574 (CXCR6). Sequence alignment was performed with Clustal Omega 1.2.2 (European Bioinformatics Institute, Wellcome Genome Campus, Hixton, Cambridgeshire, UK) [75] and visualized with ESPript 3.0 (University of Lyon, Lyon, France) [76] (both accessed on 1 February 2021).

## 3. Results

### 3.1. Homology Modeling and Validation

To investigate how cholesterol influences the behavior of CCR3, we employed Coarse-Grained Molecular Dynamics (CGMD). MD is a powerful tool to investigate protein–lipid interactions but is typically performed with a crystal, NMR, or Cryo-EM structure. Because no structure of CCR3 exists, we turned to homology modeling. We identified the reported X-ray crystal structure of the highly homologous CCR5, PDB ID 4MBS [27], as the best template candidate using the Swiss-Model template search [55]. CCR3 compared to full-length CCR5 has a sequence homology of 47%, whereas, compared to the truncated crystal structure, the % identity is 54%. CCR5 crystal structures 4MBS and 5UIW [77] displayed identical Swiss-Model Global Mean Quality Estimate (GMQE) scores of 0.71. GMQE denotes the estimated quality of a model generated from the particular template, with a maximum value of one. 4MBS was ultimately selected due to the slightly higher sequence coverage (93% vs. 92%). CCR2 is equivalently homologous but with lower GMQE scores and thus was not selected.

**Figure 1:**
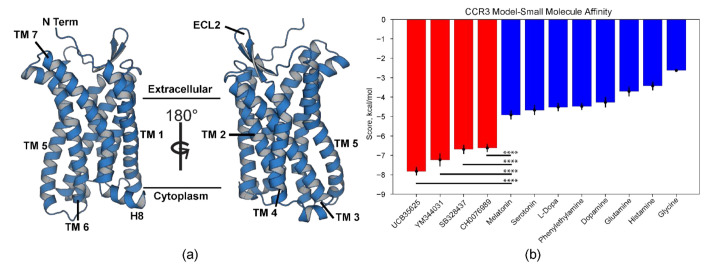
CCR3 final model and docking validation. (**a**) TM1–7, H8, and extracellular loop 2 (ECL2, ligand recognition) are highlighted on the CCR3 homology model. (**b**) Autodock experiments [36,64] between known CCR3-interacting small molecules (red) and several negative controls (blue) suggest high model quality. Data are presented as mean ± Standard Error of the Mean. Bars indicate statistical significance between the two labeled populations based on the Student *t*-test, where **** denotes *p* < 0.0001 in the Student *t*-test.

Full-length CCR3 was submitted to the Robetta comparative modeling server [54] using this structure as the template. Of the 100 models generated, the top five were displayed based on internal scoring and truncated to residues 23–317 (Figure S1). The truncated regions typically do not crystallize and therefore are untemplated during comparative modeling, generating regions of high predicted modeling error. The highest average scoring truncated model based on MolProbity scores [56], PROCHECK Ramachandran analysis [57], and ProSa [59] Z-score comparison to experimentally determined X-Ray crystallography and NMR structures was chosen (Figure S2, Table S1). We validated this structure via docking of a known CCR3 agonist (CH0076989) and several antagonists (UCB35625, YM344031, SB328437) [60,61,62] using Autodock Vina [36]. As a final step, these results were then compared to negative controls that shared some common features with the known ligands (Figure 1b). Structures for all tested small molecules are available along with ZINC database [63] identification numbers (Figure S3). The known small molecules displayed statistically significantly higher affinity compared to the selected controls. Of note, the agonist CH0076989 displayed lower binding energy than the three antagonists, which is unsurprising given that the template, and therefore our model, is of the inactive conformation. Molecules tested here share a common motif of a central amide group flanked by two bulky halogenated cyclic or aromatic groups. The most similar negative control to these small molecules was melatonin, which consists of the amide group but only a single bulky aromatic ring system. Despite this similarity, the calculated binding energy was statistically significantly lower than the lowest known ligand, CH0076989. The docking analysis in conjunction with the model scoring therefore suggests high model quality.

### 3.2. CCR3-Cholesterol Behavior in Simulation

**Figure 2:**
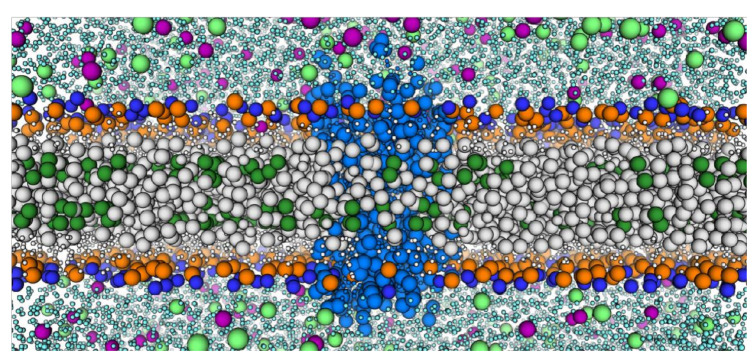
Example of the CCR3 model in a Coarse-Grained System with 30% cholesterol. CCR3 (slate blue) is surrounded by POPC (large gray beads, large dark blue and orange headgroups) and cholesterol (large green beads for the acyl tails, small white beads for the ring system). Water (cyan), sodium ions (lime), and chloride ions (purple) are represented.

We next sought to leverage this structural model to discern how cholesterol would associate with hypothetical cholesterol binding sites. To this end, we performed Course-Grained MD simulations using the Martini2.2 forcefield [49,50,51], optimized for lipid–protein interaction quantification. Here, atoms in each residue are grouped into a single backbone bead and 0–3 side chain beads [51]. We inserted our structural model into five replicates of 1-pamlitoyl-2-oleoyl-phosphatidylcholine (POPC, PC) bilayers supplemented with 0, 5, 10, 15, 20, and 30% cholesterol (*w/w*) (Figure 2). Martini2.2 treats POPC as two polar headgroup beads, two nonpolar glycerol backbone beads, and four apolar beads per lipid tail [78,79]. Cholesterol is defined with a polar -OH bead, two apolar tail beads, and a bead for each of the four rings [79,80]. The -OH and ring beads are specially parameterized to model the cholesterol ring system. These assemblies were then minimized and equilibrated before production was carried out for 10 µs at 310.15 K. Average RMSDs of each membrane composition as a function of simulation time show that a general equilibrium is quickly reached in all simulation compositions (Figure S4a). Average radial distribution functions (RDF) per composition were calculated for each lipid species, per replicate, relative to the receptor and averaged for each membrane composition. Analysis revealed a higher probability of finding cholesterol molecules around the receptor over both the expected bulk membrane cholesterol concentration (normalized to an RDF magnitude of one) and over PC (Figure S4b). Furthermore, POPC packing behavior was largely unchanged, both throughout the different simulation environments and independent of cholesterol presence.

**Figure 3:**
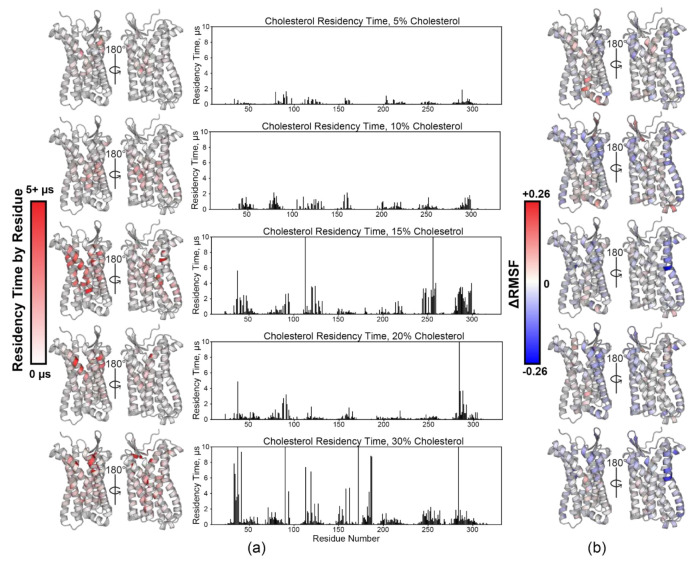
Analysis of cholesterol-titrated Coarse-Grained MD simulations. (**a**) Heatmaps of calculated cholesterol residency times per residue (left) paired with the same values plotted as a function of residue number (right) for the 5% cholesterol simulations (top) through the 30% cholesterol simulations (bottom). (**b**) Δ Root Mean Square Fluctuation (ΔRMSF) calculations reveal sites of structural plasticity (red) and rigidity (blue) mapped onto the model monomer as a function of residue number for the 5% (top) through 30% (bottom) cholesterol simulations.

Cholesterol–receptor interactions were then quantified with Pylipid [30,31,52]. Calculated cholesterol residency time by residue in general increases overall as a function of membrane cholesterol content. It is observed to blanket most of the transmembrane portion of the receptor, which is consistent with observations made from computational results of other chemokine receptors (Figure 3a) [10]. Average root mean square fluctuation (RMSF) analysis reveals a trend of increased rigidity with increasing cholesterol % (Figure 3b and Figure S5). Each simulation was compared to the 0% PC only simulations, with a more negative (blue) value indicating increased rigidity, whereas a more positive (red) value indicates increased flexibility. Enhanced rigidity is observed primarily in TM1 and TM5–7. GPCR conformational sampling inhibition by cholesterol has been observed in β_2_AR [81]; thus, we hypothesize that the same is occurring in CCR3 based on our data. Ostensibly, this would drive conformational equilibrium towards a state that will more favorably bind the ligand. In the 15% simulation, we observed an inflection point in both cholesterol residency time and ΔRMSF.

### 3.3. Pylipid Predicts Cholesterol Binding Sites in CCR3 Populated with CRAC and CARC Motifs

**Figure 4:**
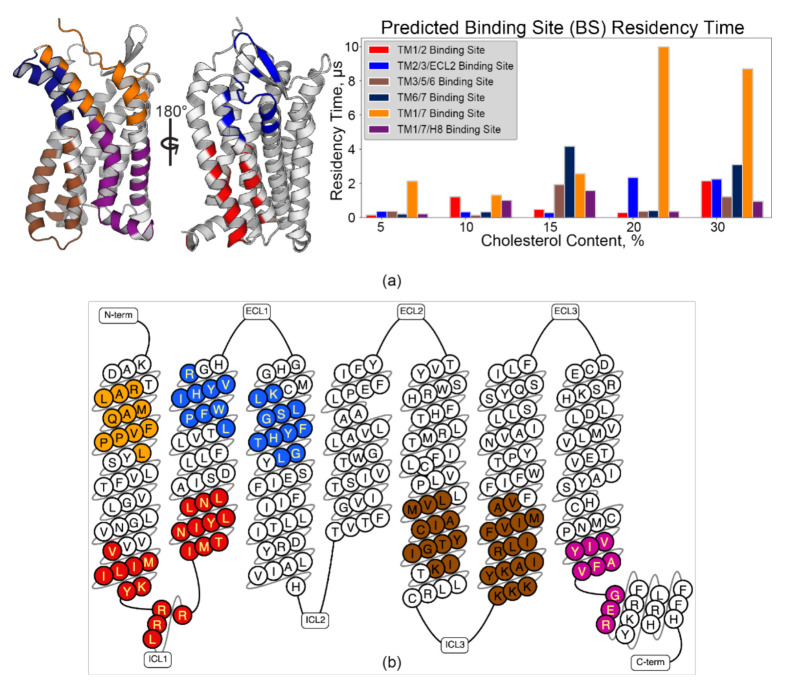
Pylipid-predicted cholesterol binding sites are conserved across membrane conditions and populated with CRAC/CARC motifs. (**a**) Binding sites are mapped onto the model (left), with cholesterol residency time plotted as a function of membrane cholesterol % (right) for binding sites identified in grooves between TM helices. (**b**) CRAC/CARC motifs are color-coded by which predicted binding site they are found within, produced using the GPCRdb [74].

Pylipid-predicted binding sites were more or less equivalent across membrane compositions (i.e., the predicted sites contain the same core residues), though some site swapping of peripherally related residues was observed (Figure 4a and Figure S6). Nonetheless, broadly defined binding sites conformed to previous computational studies of chemokine receptors (discussed below). This implies non-stochastic packing around the receptor, which corroborates observations made from RDF data. The grooves between extracellular (EC) sides of TM1/7 (Figure 4, orange) and TM6/7 (Figure 4, dark blue) may be functionally crucial based upon the residency time predicted by Pylipid analysis. Moreover, the 10 µs and 8.7 µs residency times for 20% and 30% cholesterol, respectively, promote the TM1/7 site as a candidate for future experimental interrogation. Identification of Cholesterol Recognition/Interaction Amino Acid Consensus (CRAC, L/V–X_1–5_–Y–X_1–5_–R/K) motifs [14,15], or the reverse, CARC motifs (R/K–X_1–5_–Y/F–X_1–5_–L/V) [15,16], is a typical starting point for cholesterol binding site identification. CCR3 contains four CRAC sites and eight CARC sites (Figure S7a,b). Interestingly, all of the populated binding sites *except* TM6/7 contained at least one CRAC/CARC motif, comprising 9 of the 12 sites found within CCR3 (Figure 4b). The remaining sites (Figure S7c) sorted into EC and intracellular (IC) Pylipid-predicted binding sites integrating components from TM3, 4, and 5. However, cholesterol residency in those sites was statistically insignificant relative to simulation noise (Figure S8). R202^5.41^ to L208^5.47^, which were highly rigid according to ΔRMSF calculations, existed within the TM3/4/5 EC site (dark teal) but did not strongly interact with cholesterol.

### 3.4. CARC-Like Motif in the TM1/7 Binding Site

**Figure 5:**
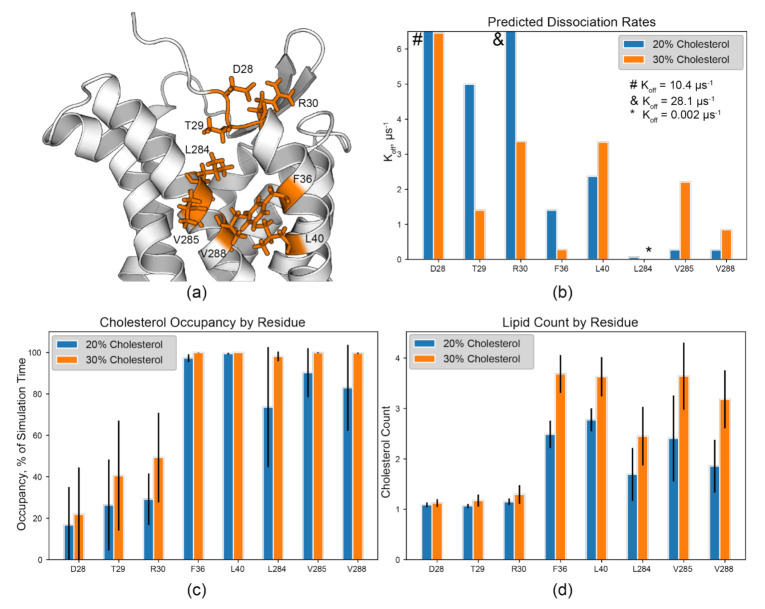
The TM1/7 site as a potential CCR3 cholesterol binding site. (**a**) CARC and CARC-adjacent residues identified in the TM1/7 binding site, color coded as in Figure 4. R30^1.28^–F36^1.34^–L40^1.38^ form the CARC motif in this binding site. (**b**) Predicted K_off_ values, (**c**) lipid occupancy as a percent of simulation time, and (**d**) lipid count from the 20% and 30% cholesterol simulations. Error bars indicate the standard deviation between replicates. Symbols (#, &, and *) denote values outside of the plot view.

The binding site between EC TM1 and 7 is perhaps a special case based on the calculated residency time (Figure 4, orange). Here, the CARC sequence of R/K–X_1–5_–Y/F–X_1–5_–L/V [17] is identified in TM1 as R30^1.28^–F36^1.34^–L40^1.38^ (Figure 5a and Figure S7b,c). The residues L284^7.36^, V285^7.37^, and V288^7.40^ in TM7 are proximal to this motif. Their sidechain methyls likely contribute to the hydrophobic groove between these two helices and are found within this predicted binding site. Further, these residues exhibit relatively low K_off_ values, high cholesterol occupancy, and high lipid count at 20% and 30% cholesterol content (Figure 5a,c). L284^7.36^ has a K_off_ of 0.067 µs^−1^ at 20% and 0.002 µs^−1^ at 30%.

Lipid count analysis shows that multiple cholesterols occupy this site throughout the majority of the simulation time at greater than 15% cholesterol content, correlating to the greater occupancy times for these hydrophobic residues (Figure 5c,d). Simultaneous occupation of this site is likely, and therefore F36^1.34^ may participate in π stacking while aliphatic, branched residues in both TM1 and 7 form hydrophobic contacts with cholesterols as the molecules are moving within the site. R30^1.28^ of the identified CARC site likely forms a hydrogen bond with the hydroxyl group of cholesterol, but ambiguous K_off_ values for R30^1.28^ (µs regime) hint that hydrogen bond formation may be mediated or transient (Figure 5b). However, hydrogen bond formation between cholesterol and protein may not be limited to R and K residues [15]. The sidechains of D28^1.26^ and T29^1.27^ are both positioned to form hydrogen bonds with cholesterol molecules within this groove (Figure 5a,b). However, it is unknown if this exists in vivo and this predicted binding site should be targeted experimentally. Regardless, tight binding is evident for F36^1.34^, L40^1.38^, L284^7.36^, V285^7.37^, and V288^7.40^. Similar contributions to cholesterol binding were observed in the serotonin receptor 5-HT_1A_, where residues L^7.32^ and I^7.36^ in TM7 played a critical role in cholesterol binding in this site [82]. Thus, we hypothesize that the CARC motif and surrounding branched aliphatic residues play a central role as the core of a cholesterol binding site occupied by multiple cholesterols in CCR3.

### 3.5. Rationaliztion of Identified CRAC/CARC Motif Conservation in Other Chemokine Receptors

**Figure 6:**
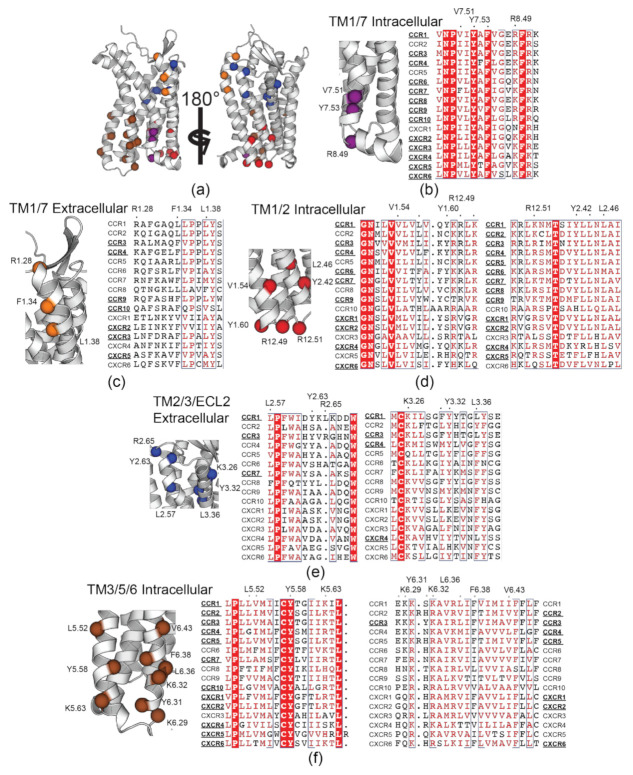
Conservation of CCR3-identified Cholesterol Recognition/Interaction Amino Acid Consensus motif (CRAC) and its inversion (CARC) motifs in other CC and CXC chemokine receptors. (**a**) Overview of CRAC/CARC residues in Pylipid-predicted binding sites, zoomed and aligned in (**b**) TM1/7 intracellular side, (**c**) TM1/7 extracellular side, (**d**) TM1/2, (**e**) TM2/3/ECL2, and (**f**) TM3/5/6. Each residue is color coded by which binding site it resides in. Residues above alignments indicate the CCR3 residue in the CRAC or CARC motif and its position according to the Weinstein–Bellesteros numbering convention [87]. Dots near labeled residues represent other amino acids that could feasibly satisfy the cholesterol motif sequence. Bolded and underlined receptor names indicate conservation of the motif in the receptor, though the residue position may fluctuate. In (**f**), there are two motifs in TM6 (right). Bolded and underlined receptors on the left of the alignment indicate the site containing Y^6.31^, while the right contains F^6.38^.

The cholesterol recognition motifs identified in CCR3 are not isolated. Indeed, the residues that comprise these motifs are largely conserved in other CC and CXC motif chemokine receptors (Figure 6). Unsurprisingly, functional residues in and near these sites are implicated in receptor dimerization, ligand binding, and signal transduction, often with complicated interplay between all three.

The TM1/7 IC binding site (Figure 6b) was previously identified as a putative cholesterol binding site in CXCR4 and CCR5 [11]. Further sequence alignment reveals that this site is largely conserved throughout the entire subclass. Conservation of this site is not surprising given that the aromatic core Y^7.53^ is from the NPxxY motif, which plays a critical role in the inactive–active conformational exchange [83]. Additionally, there is considerable overlap between the putative residues identified in CXCR4 and CCR5 and the TM1/7 IC binding site observed in this work (Figure 4 purple and Figure S9). Here, it is apparent that the residues identified in that work are largely conserved in all CC and CXC motif receptors, not just CCR3. Surprisingly, the TM1/7 EC site is not well conserved (Figure 6c) despite high cholesterol residency in simulations of CCR2 and CCR5 [10]. It is unclear whether the presence of the CARC motif here is CCR3-specific or if it may play a broader role in chemokine receptor biology.

The TM1/2 site is located spatially close to D80^2.50^. D^2.50^ serves two functional roles. During signal transduction, conformational cascades originating from the orthosteric ligand pocket travel to the toggle switch residue W^6.48^, then D^2.50^, and finally to intracellular repacking that would accommodate the G protein [84]. Both residues also coordinate allosteric Na^+^ in an inactive-like state [85,86]. It is therefore no surprise that cholesterol as an allosteric modulator would find residence near these key signal transduction residues. The proximity of the TM6/7 binding site (Figure 4, dark blue) to W252^6.48^ may therefore be significant for a similar reason, though it lacks a CRAC or CARC motif. Overall, this provides a reasonable explanation for the high conservation of these TM1/2 CRAC/CARC motifs in other chemokine receptors (Figure 6d).

The pattern of cholesterol-induced receptor rigidity in TM5 measured via ΔRMSF (Figure 3b) mimics binding site residency time in the TM3/5/6 binding site (Figure 4, brown). Thus, we conclude that it is cholesterol occupancy in this site that drives the observed increase in rigidity. Of note, P211^5.50^ in the P211^5.50^–I121^3.40^–F248^6.44^ motif is directly adjacent to this binding site [88]. This cluster of residues acts as a ‘connector’ motif reported to link ligand binding at the agonist binding pocket to the NPxxY motif in TM7 during activation [89,90,91]. The TM2/3/ECL2 binding site follows a similar trend, leading to decreased flexibility in ECL2 (Figure 3b), which is heavily involved in chemokine ligand recognition [92]. These CARC sites are also located spatially nearby I121^3.40^ in the PIF motif and D80^2.50^. In concert, cholesterol in these two binding sites may therefore play a role in cholesterol-driven conformational selection, which is hypothesized to be a contributing factor to cholesterol-induced changes in ligand affinity [93]. However, conservation of TM2/3/ECL2 CARC sites (Figure 6e) is relatively poor compared to TM3/5/6 (Figure 6f) and may therefore play a secondary or CCR3-specific role to the highly conserved CRAC and CARC motifs in the TM3/5/6 binding site.

## 4. Discussion

### 4.1. Cholesterol and Conformational Sampling

The influence of cholesterol on GPCR conformational dynamics was previously documented [93]. In our simulations, it is possible that the nonlinearity of overall cholesterol binding site residency arises due to concentration-specific conformational sampling whereby cholesterol facilitates the exclusion of conformations energetically unfavorable for ligand-binding. We hypothesize that the concentration of general residency time in the 20% replicates (~13 µs total time) to the TM1/7 EC and TM2/3/ECL2 sites may be due to this concentration-dependent preference. This is in juxtaposition to the 15% analysis (~10.5 µs total residency time) whereby the residency time is spread over four or more binding sites. We predict that this, in the context of the observed residency time and ΔRMSF patterns, indicates a direct competitive allosteric cholesterol–CCR3 interaction.

In our simulations, we observe that TM5–7 are prominent sites of increased rigidity. Specifically, residues R202^5.41^ to L208^5.47^ in TM5 display a significant loss of flexibility. However, these residues are not predicted to be involved in any cholesterol binding sites of note (Figure S8, dark teal). TM5–7 are involved in repacking during Class A GPCR activation, and mutations of key residues in them strongly affect activity, typically by influencing the inactive–active state transition as discussed above [94]. The EC sides of these helices also form the bulk of the orthosteric ligand pocket in Class A GPCRs, in conjunction with TM3 [93]. Kink residues P^5.50^ in the PIF motif, P^6.50^, and P^7.50^ in the NPxxY motif in these helices are essential for coupling of ligand-binding to global conformational changes observed during receptor activation. Thus, we hypothesize that the increasing rigidity of this region through allosteric cholesterol binding prepares the orthosteric pocket to receive the ligand and shifts equilibrium towards a conformation that will be more easily activated by the ligand for signal transduction.

### 4.2. Curerent Experimental Evidence of CRAC/CARC Occupancy in Chemokine Receptorss

While most GPCR structures containing cholesterol appear to favor the CCM, several are found to have CRAC or CARC occupancy [24]: cannabinoid receptor 1 (CB_1_) (6N4B) [95], the cysteinyl leukotriene receptor 2 (6RZ7) [96], and the formyl peptide receptor 2 (6LW5) [97]. There is therefore direct structural evidence of CRAC and CARC occupancy in Class A GPCRs. To date, however, CCR9 (5LWE) [26] and CXCR2 (6LFM) [29] are the only chemokine receptor structures found to contain any cholesterol. In CCR9, cholesterol localizes to what is referred to as an EC TM6/7 CCM, conspicuously located in space overlapping with the TM6 CARC motif (Figure 6f, far right) and the TM7 CRAC motif (Figure 6b), though we draw no conclusions from this overlap. In CXCR2, cholesterol localizes to a CRAC variant in TM2/3/4 characterized by a Trp residue as the aromatic core of the binding site. Additionally, the previously identified putative cholesterol binding site in CXCR4 and CCR5 [11] was accurately predicted here in our own simulations (Figure S9).

Beyond these few examples, very little is to be found concerning cholesterol binding sites in chemokine receptors beyond whole helical contact interfaces in dimerization discussed below. Nevertheless, many chemokine receptors are shown to be sensitive functionally to membrane cholesterol, typically deciphered through cholesterol sequestration [12]. We find it highly unlikely that chemokine receptors as a group would not evolve conserved mechanisms of cholesterol sensitivity. More information is naturally required to draw significant conclusions, but we suggest here that the extrapolation of CCR3 binding site data to other chemokine receptors is not erroneous given that our data also accurately predicted residency in the putative CXCR4/ CCR5 site in the inner leaflet of TM1 and 7.

### 4.3. Cholesterol as Dynamic Glue Driving Multimerization

Ultimately, cholesterol-driven dimerization of CCR3 is beyond the scope of this work. However, a discussion over cholesterol–receptor interaction is nonetheless incomplete without mention of what role these binding sites may play in the phenomenon of receptor multimerization. It is known that CCR3 forms functional dimers and higher-order oligomers, but the role of cholesterol in this phenomenon is unknown [98]. Therefore, the binding sites identified in this work should be considered during future evaluation of CCR3 homo- or hetero-dimerization sites.

It was noted above that the TM1/7/H8 site has already been identified in a number of other chemokine receptors. In the case of the EC TM1/7 site, a previous computational study identified this region as a ‘lipid entry portal’ in both CCR2 and CCR5, with relatively high cholesterol density on TM1 and TM7 residues in this region [10]. Interestingly, their study also resulted in high density in the groove between the TM6/7 EC side for both receptors and some density in the TM1/7/H8 site on CCR2. Ultimately, it was observed that for both CCR2 and CCR5, TM1 was a homodimerization hotspot in silico, facilitating homodimerization through contacts of TM1/H8 and TM1/H8, TM1/H8 and TM4/5, and TM1/H8 and TM5–7, though a number of less populated dimer interfaces were observed. Lastly, the observed dimer interfaces in their work identified interfaces distinct from those derived from CXCR4 in a prior study, inferring that CC motif receptors form dimers in ways separate from CXC motif receptors [13].

Another study found that the TM3/4/5 and TM5/6 interfaces play a pivotal role in CCR5 homodimerization in vitro and in vivo [99], while the crystal structure helical contact site in complex with the anti-retroviral maraviroc appears to be between TM1/7 of one monomer and TM3/4/5 of the other [27]. In aggregate, these observations suggest a broadly defined swath of helical real estate involved in identified dimer interfaces in CC motif chemokine receptors, corroborated by the observed patterns in cholesterol residency predicted for CCR3 in this work. It is known that CCR3 forms dimers and higher-order oligomers in vivo [98], that dimer formation can influence receptor–ligand affinity [100], and that cholesterol typically drives dimer formation in chemokine receptors [10,13]. Thus, the binding sites identified herein will also serve as a starting place to explore the dimerization interfaces of CCR3.

### 4.4. Pitfalls in Attempted Sorting of Cholesterol Binding Sites: Rejection of the Bear Thesis

A recent study made use of Consensus Network Analysis to sort the probability of binding based on cholesterol density in 473 X-ray crystal and Cryo-EM structures [21]. The authors found that 92% of cholesterol located to 12 clusters and concluded that there were no broadly recurring cholesterol binding motifs in GPCRs. This is not particularly surprising as the supposedly predominant cholesterol binding site, the cholesterol consensus motif, is only found in 21% of Class A GPCRs [6]. It has also been independently noted that just because a certain motif exists does not mean that it will be occupied [25]. We have observed this as several CARC motifs in CCR3 were not found to have any considerable cholesterol occupancy in our simulations (Figure S8).

Evolutionary drift of conserved residues is reported even within the CC motif family [101,102]. MD analysis of different chemokine receptors also suggests favored interaction sites that are not necessarily equivalent from receptor to receptor [10]. This would seem to further suggest that classification is indeed pointless, but we instead offer an alternative possibility. While the lack of cohesion in sites observed thus far is frustrating, it is not inconceivable that it would arise from conservation within subsets within each family. This could then explain why the CRAC and CARC motifs observed herein, as well as other sites observed in the literature, such as the CCM in β_2_AR [6], are sometimes conserved and sometimes not. It is evident that further analysis is required, which we certainly find to be a worthwhile pursuit. Thus, we consider the binding sites identified herein to be possibilities not just within CCR3 but chemokine receptors in general. This does not, however, preclude the need for direct validation in any receptor that may appear to conserve a given motif.

## 5. Conclusions

We have generated and validated a homology model of CCR3 based on the CCR5 crystal structure. This model was leveraged to identify cholesterol binding sites from CGMD simulations in a manner similar to other membrane protein systems [30,31,32,33,34,35]. It was noted that the Pylipid-predicted binding sites contained most of the CRAC and CARC motifs in CCR3. Using sequence alignment, we show that many of these sites are conserved in other chemokine receptors. The binding sites predicted herein are suggested as possibilities which will be used to direct our future work with CCR3 in vitro and could serve the same purpose for other chemokine receptors.

## Data Availability

Data is available upon request from corresponding author.

## Supplementary Materials

The following are available online at https://www.mdpi.com/article/10.3390/membranes11080570/s1, Table S1: Model MolProbity Scores, Table S2: Docking Parameters, Figure S1: CCR3 Models and Modeling Error, Figure S2: CCR3 Model 1 Validation and Comparison to CCR5 Template, Figure S3: Small Molecules, Figure S4: RMSD and RDF Analysis, Figure S5: RMSF Analysis, Figure S6: Pylipid-Predicted Cholesterol Binding Sites, Figure S7: Identification of CRAC and CARC Motifs in CCR3, Figure S8: Low Residency CARC Sites, Figure S9: Noncanonical Putative Cholesterol-Binding Site.
