# Supplementary material for: In Silico Identification of Cholesterol Binding Motifs in the Chemokine Receptor CCR3
Source: Membranes (Basel). 2021 Jul 28;11(8):570. doi: 10.3390/membranes11080570 (PMC8401243; doi:10.3390/membranes11080570)
Supplement: Supplementary file 1 [file membranes-11-00570-s001.zip › membranes-1278703-supplementary.pdf]

membranes

Supplementary Information

In Silico Identification of Cholesterol Binding Motifs in the Chemokine Receptor CCR3

Evan van Aalst, Jotham Koneri and Benjamin J. Wylie *

Department of Chemistry and Biochemistry, Texas Tech University, Lubbock, TX 79423, USA; Evan.van-Aalst@ttu.edu (E.v.A.); Jotham.Koneri@ttu.edu (J.K.)
* Correspondence: Benjamin.J.Wylie@ttu.edu

Citation: van Aalst, E.; Koneri, J.; Wylie, B.J. In Silico Identification of Cholesterol Binding Motifs in the Chemokine Receptor CCR3. *Membranes* **2021**, *11*, 570. https://doi.org/10.3390/membranes11080570

Academic Editors: Yosuke Senju and Shiro Suetsugu

Received: 13 June 2021
Accepted: 22 July 2021
Published: 28 July 2021

Publisher's Note: MDPI stays neutral with regard to jurisdictional claims in published maps and institutional affiliations.

Copyright: © 2021 by the authors. Licensee MDPI, Basel, Switzerland. This article is an open access article distributed under the terms and conditions of the Creative Commons Attribution (CC BY) license (http://creativecommons.org/licenses/by/4.0/).

Table S1. MolProbity [1] analysis of CCR3 Homology Models truncated to residues 23–317. *** denotes the selected model. The CCR5 template PDB ID 4MBS is included for comparison [2].

	Model 1***	Model 2	Model 3	Model 4	Model 5	CCR5 (4MBS)	Goal
Clashscore	1.22	1.42	1.02	1.22	1.02	2.47	
Poor Rotamers	1 (0.38%)	1 (0.38%)	0	0	1 (0.38%)	28 (4.84%)	<0.3%
Favored Rotamers	261 (98.49%)	261 (98.9%)	262 (98.49%)	261 (98.49%)	262 (98.49%)	506 (87.54	>98%
Ramachandran Outliers	0	0	1 (0.34%)	1 (0.34%)	1 (0.34%)	6 (0.87%)	<0.05%
Ramachandran Favored	286 (97.61%)	282 (96.25%)	286 (97.61%)	284 (96.93%)	286 (97.61%)	664 (96.51%)	>98%
Rama. Distribution Z-score	−0.25 ± 0.45	−0.38 ± 0.44	0.79 ± 0.47	0.70 ± 0.49	0.06 ± 0.46	−1.29 ± 0.29	ǀZ scoreǀ<2
MolProbity Score	0.92	1.13	0.88	1.02	0.88	1.78	
Cβ deviations >0.25Å	0	0	2 (0.71%)	0	1 (0.35%)	0	0%
Cα Geometry Outliers	0	2 (0.69%)	0	0	2 (0.69%)	2 (0.29%)	<0.5%
Bad bonds	2/2493 (0.08%)	5/2493 (0.2%)	4/2493 (0.16%)	0/2493	0/2493	0/5641	0%
Bad angles	7/3391 (0.21%)	14/3391 (0.41%)	8/3391 (0.24%)	16/3391 (0.47%)	15/3391 (0.44%)	20/7698 (0.26%)	<0.1%
Cis Prolines	0/9	0/9	1/9	0/9	0/9	0/26	≤1 per chain, ≤5%
Twisted Peptides	0	0	0	0	2 (0.68%)	0	0

Table S2. Docking Parameters used in AutoDock Vina [3] analysis. .

Receptor	Ligand	center_x	center_y	center_z
CCR3_model.pdbqt	ligand.pdbqt	152.076	117.239	28.933
energy_range	exhaustiveness	size_x	size_y	size_z
4	8	104	102	50

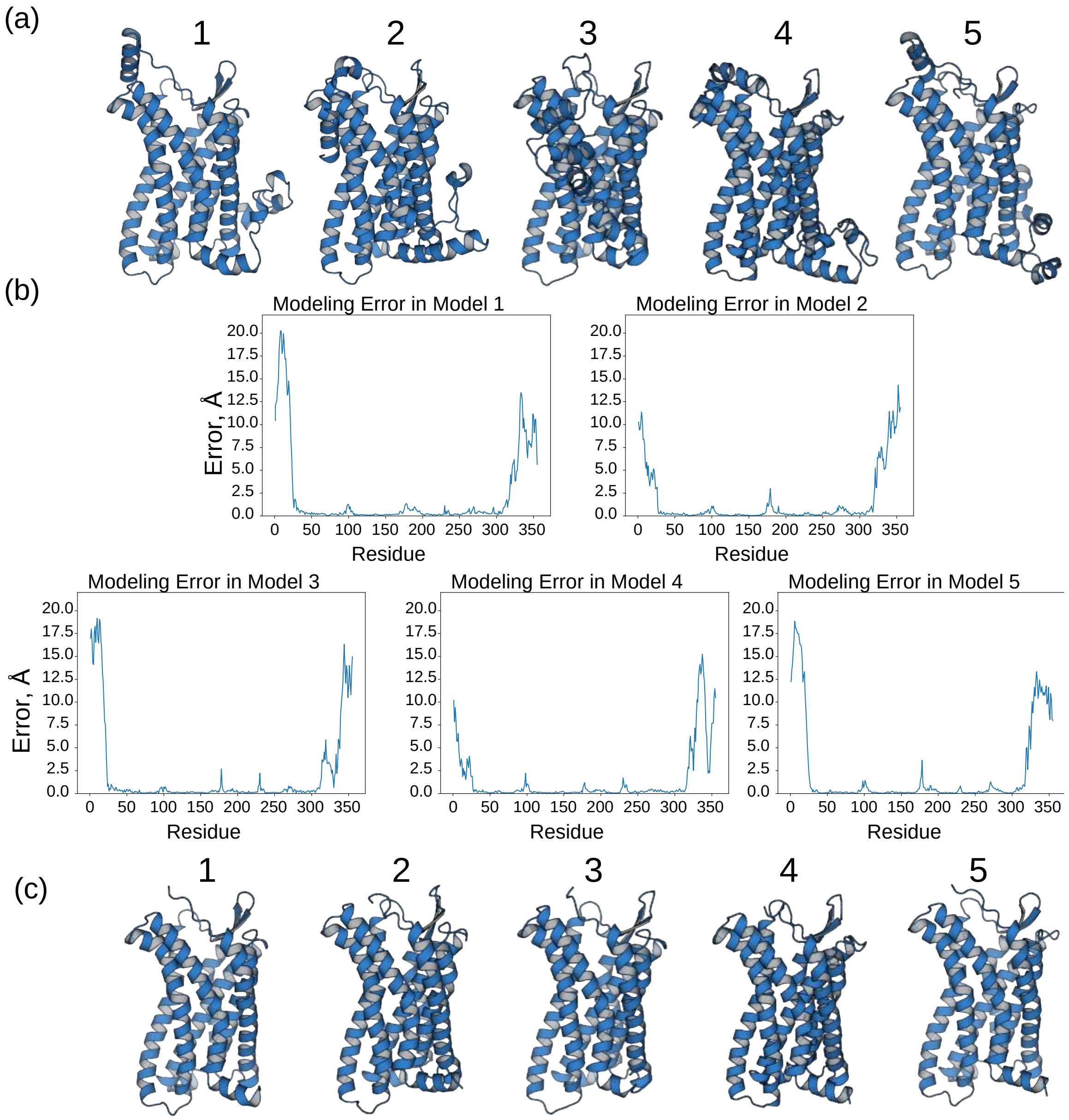

Figure S1. CCR3 model generation and validation. (**a**) 5 full-length models generated by Robetta (1–5 left-right) [4]. (b) Modeling error per residue associated with each model, estimated by Robetta. (**c**) Models in (**a**) truncated to residues 23–317 (1–5 left-right) to forego error-prone regions identified in (**b**). Model 1 was selected for this work.

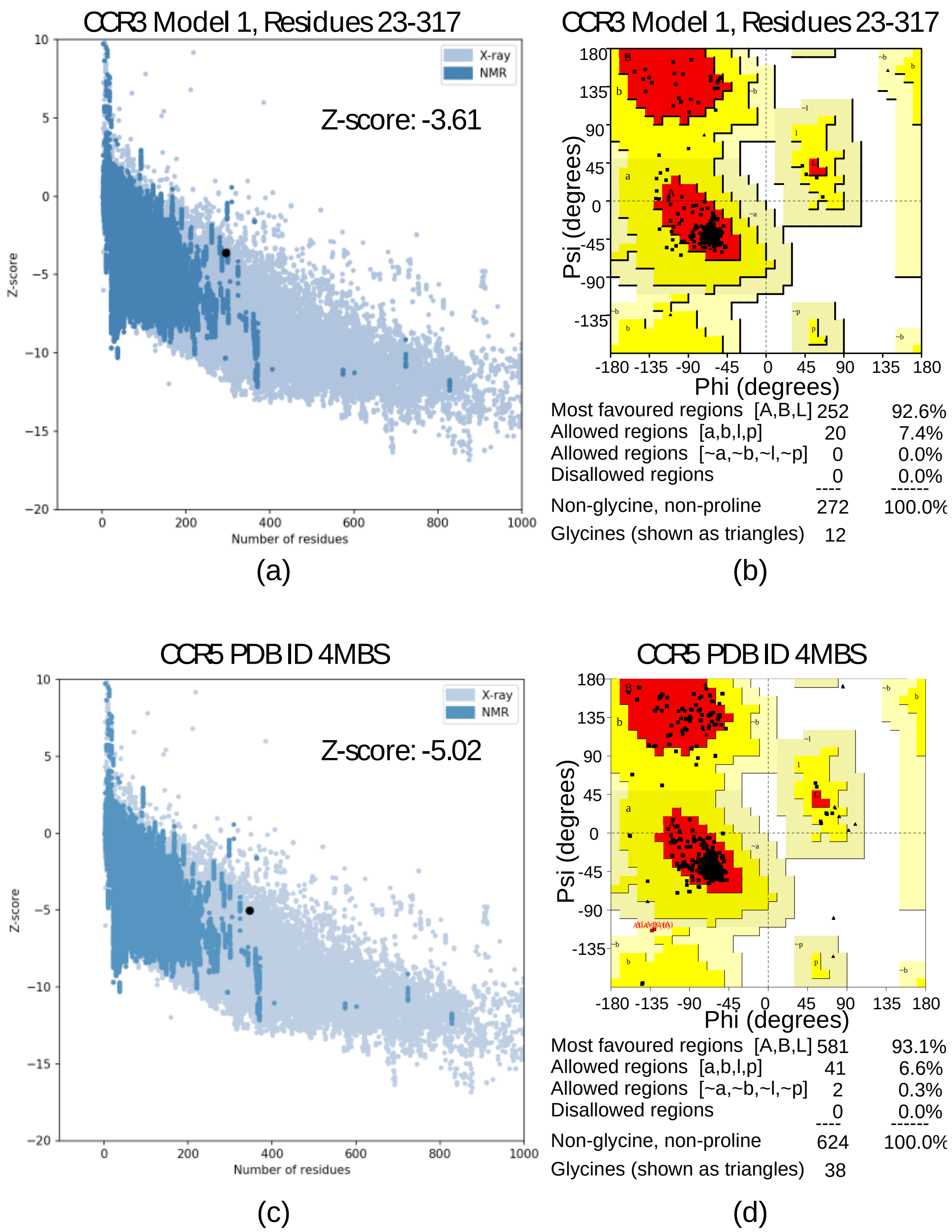

Figure S2. CCR3 model validation, compared to the CCR5 template 4MBS. (**a**) ProSa Z-score [5] as a function of total residue number for the CCR3 Homology Model Chosen for this work. The black dot represents the ROBETTA CCR3 homology model. A Z-score of -3.61 falls within the Z-score range of experimentally determined conformations as determined by NMR (dark blue) and X-Ray Crystallography (light blue) of similar number of residues. (**b**) Ramachandran analysis of the CCR3 model chosen for this work using PROCHECK [6]. (**c**) Z-score comparison and (**d**) Ramachandran analysis for the CCR5 template used in this work for comparative modeling.

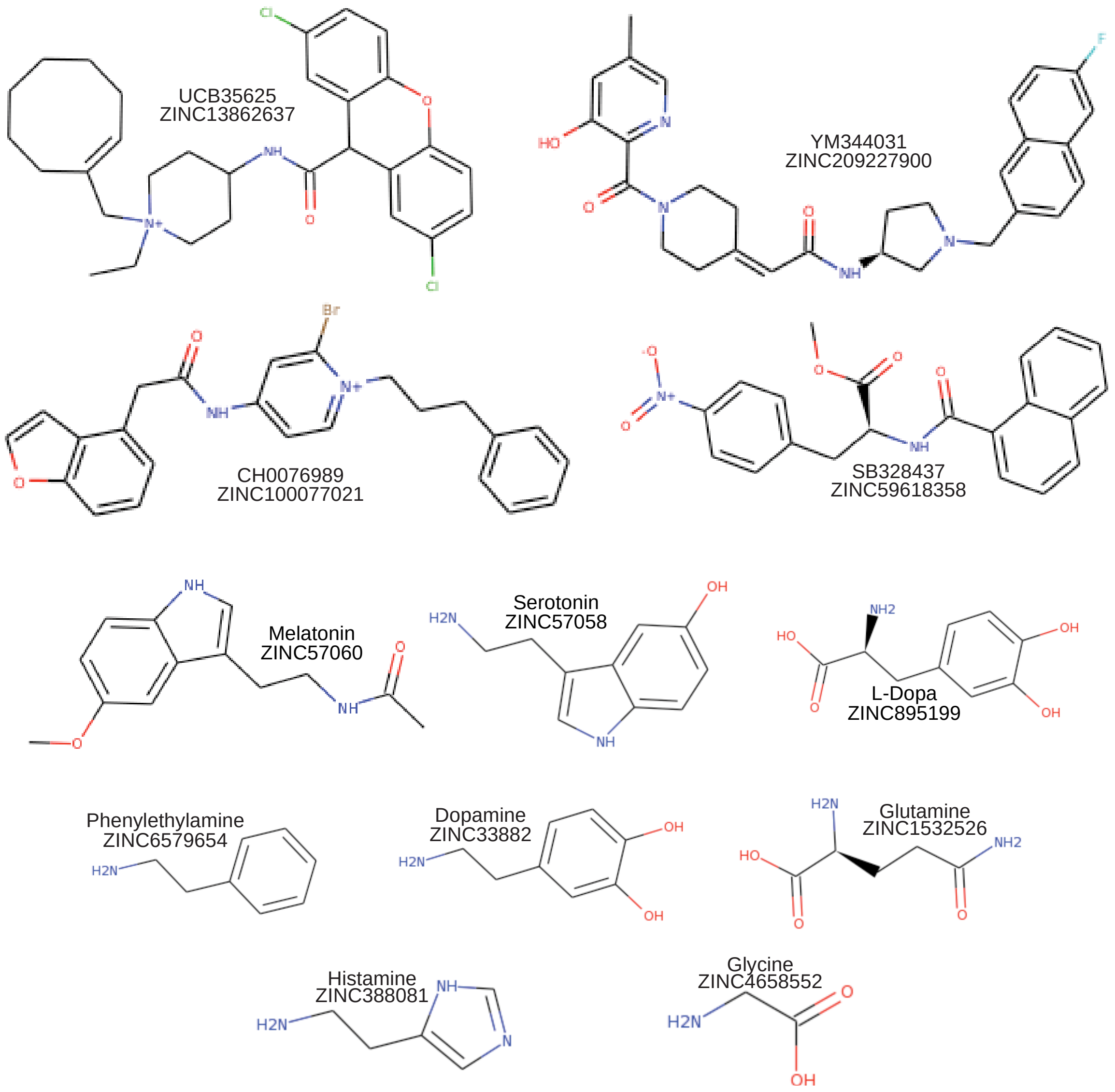

Figure S3. Structures, names, and database substance identification numbers (ZINC database [7]) of the small molecules docked to the CCR3 model.

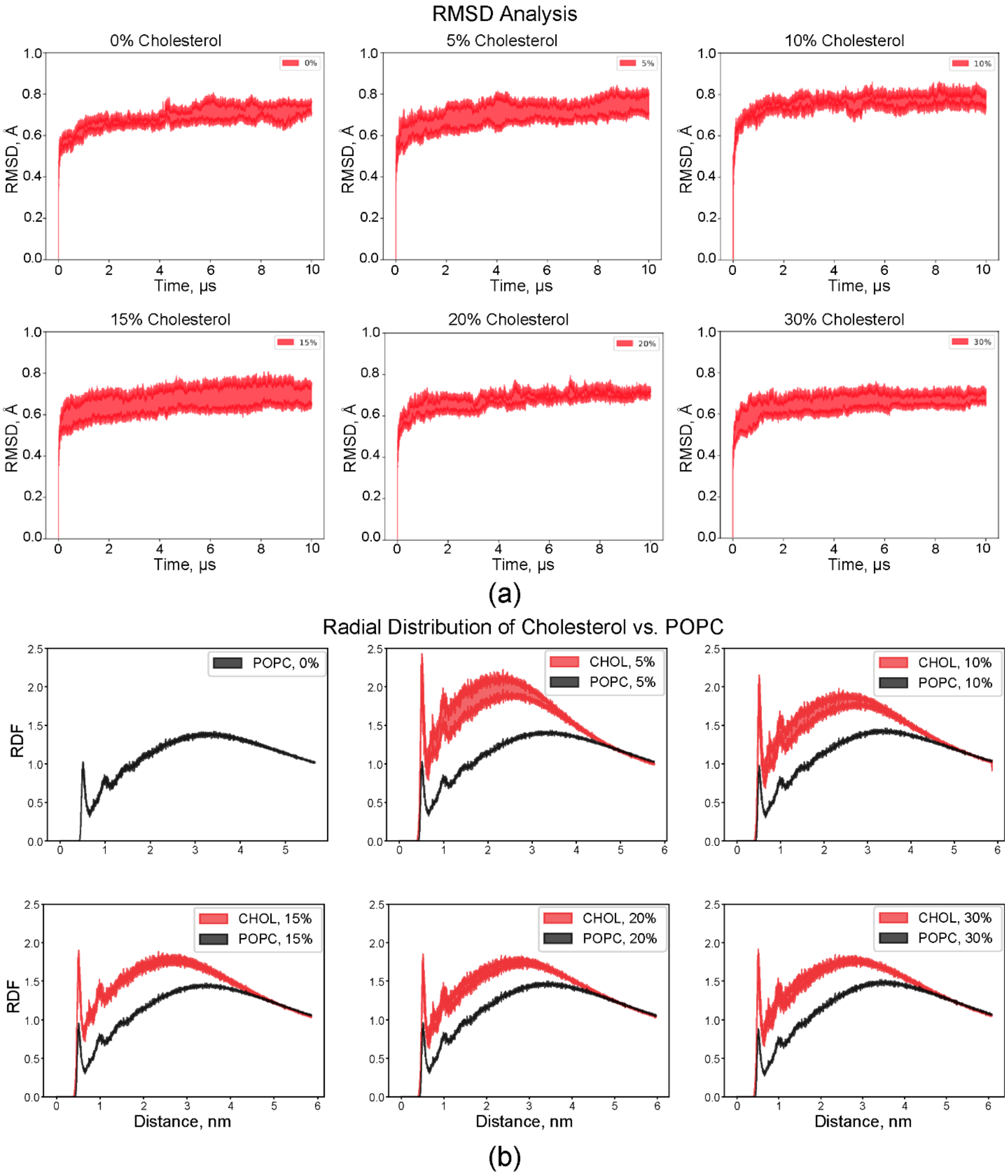

Figure S4. Average RMSD and RDF analysis of replicates for simulation runs. (**a**) RMSD analysis suggests a general equilibrium is quickly reached. (**b**) Results suggest cholesterol preferentially packs around the receptor over PC, evidenced by higher calculated cholesterol RDFs within ~4 nm. Both PC and cholesterol RDF values trend to 1 (no correlation) shortly after 4 nm. Line thickness is correlated with increased variance in standard deviation among the five replicates for each membrane composition. The PC RDF behaves the same in all simulations. The cholesterol RDF follows a gradually tightening of variance, suggesting variable packing at lower percent membranes.

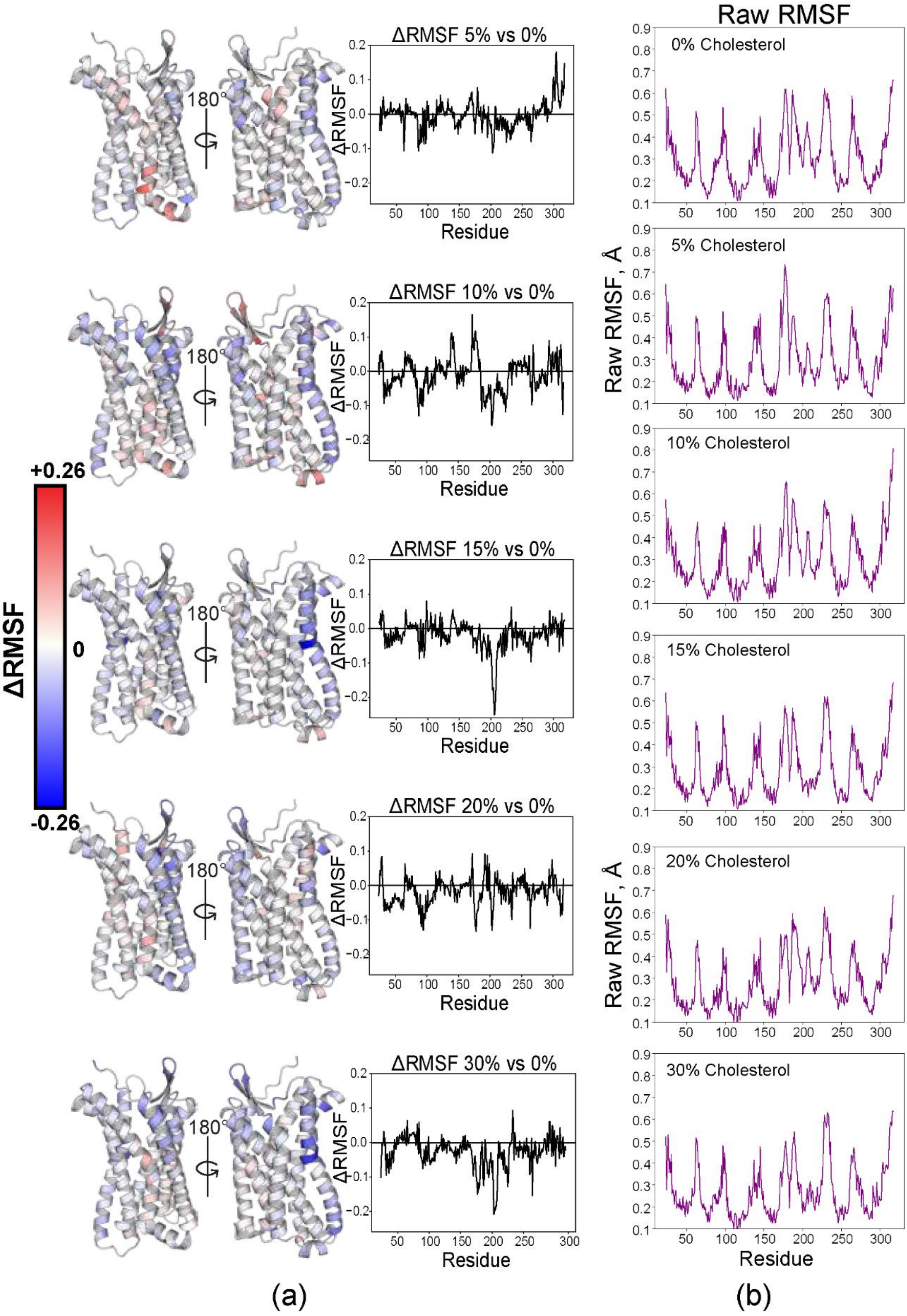

Figure S5. RMSF analysis of simulation data. (**a**) ΔRMSF mapped onto the monomer (left) and plotted (right) for each cholesterol composition. Increasing rigidity (blue) suggests cholesterol-driven conformational selection. (**b**) Raw RMSF values used to calculate ΔRMSF in (**a**).

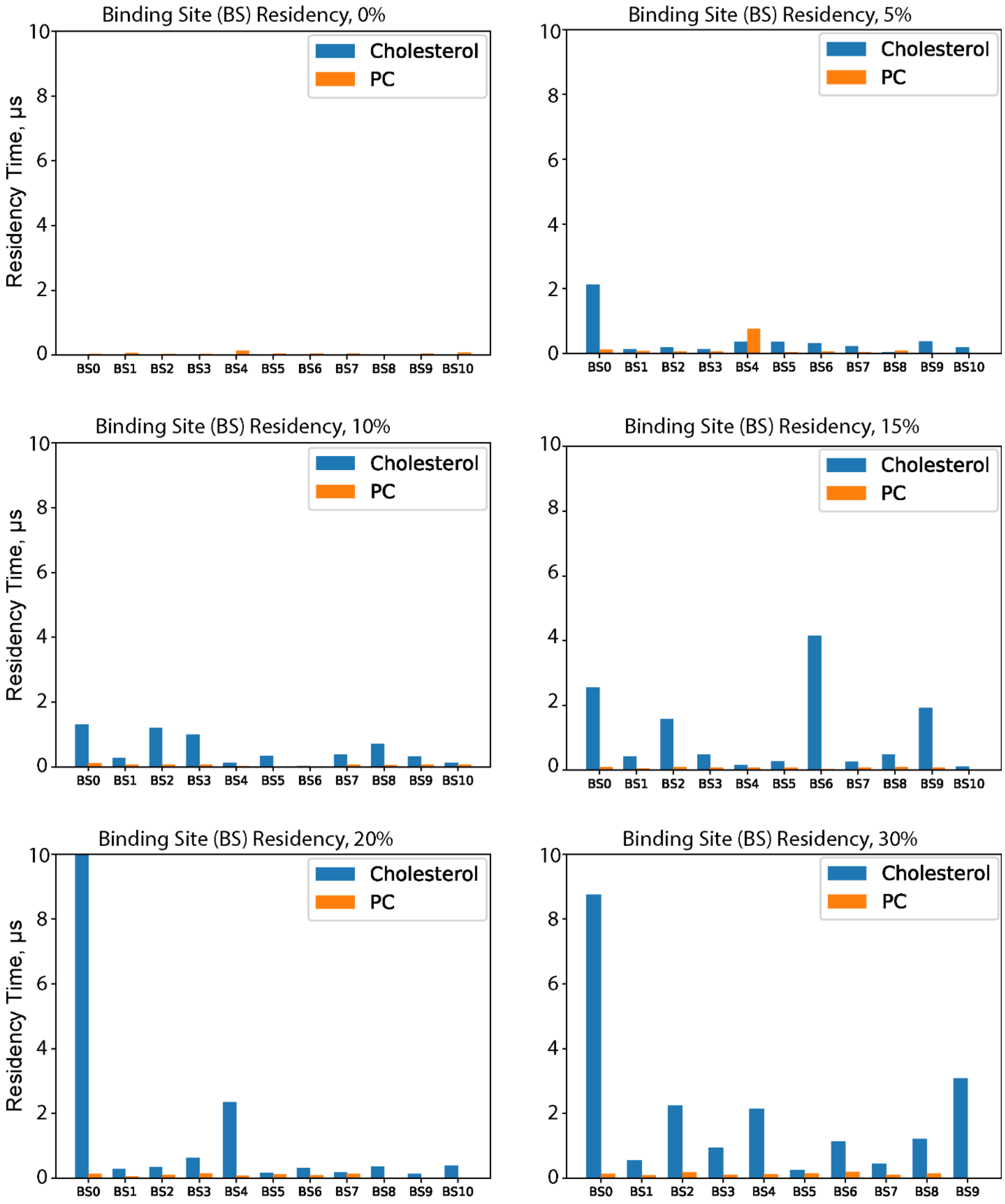

Figure S6. Plotted Pylipid-predicted [8–10] binding sites for both cholesterol and PC, for all runs. Binding site numbering is based on the first residue sequentially in the primary sequence and does not necessarily reflect the same binding site in different runs. Cholesterol is preferentially interacting with the receptor in each average of simulations; PC behavior is both consistent and non-interacting.

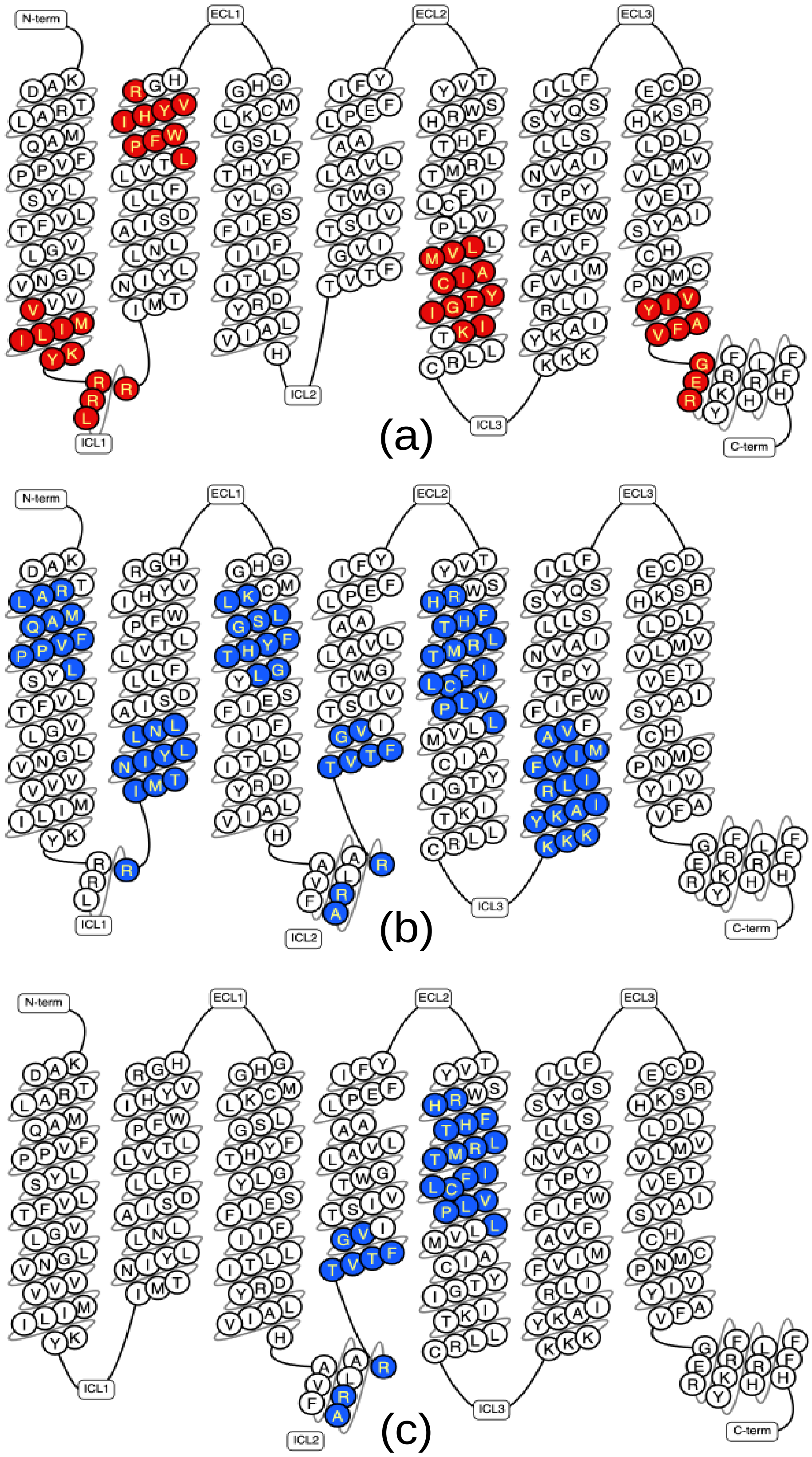

Figure S7. Identification of Chemokine Receptor Cholesterol-Binding Sites. (a) CRAC motif [11,12] (L/V–X_{1-5}–Y–X_{1-5}–R/K) sequences are highlighted in red. (b) CARC motif [12,13] (R/K–X_{1-5}–Y/F–X_{1-5}–L/V) sequences are highlighted in blue. Sequences were derived from Fantini and Barrantes, 2013 and mapped onto snake plots using the GPCR database [14]. (c) Motifs in CCR3 lacking cholesterol residency time as identified by PyLipID [8,9], color coded as in (a) and (b).

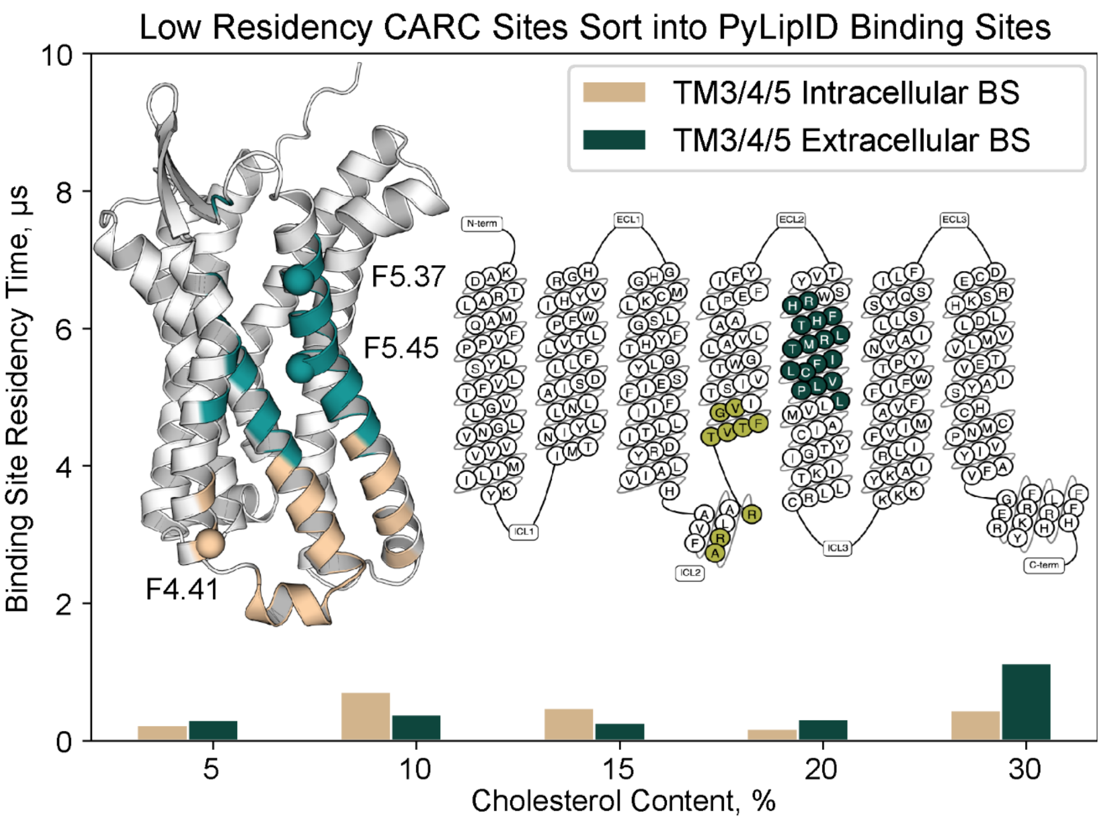

Figure S8. Several CARC sites [12,13] found within PyLipID-predicted binding sites [8,9] of unimpressive cholesterol residency time. Cholesterol residency time in the TM3/4/5 extracellular (dark teal) and intracellular (tan) sites is quite low. CARC site Phenylalanines are labeled on the left (Weinstein-Ballesteros numbering [15]), and the full sites are visualized on the right in snake plot form (GPCRdb, [14]).

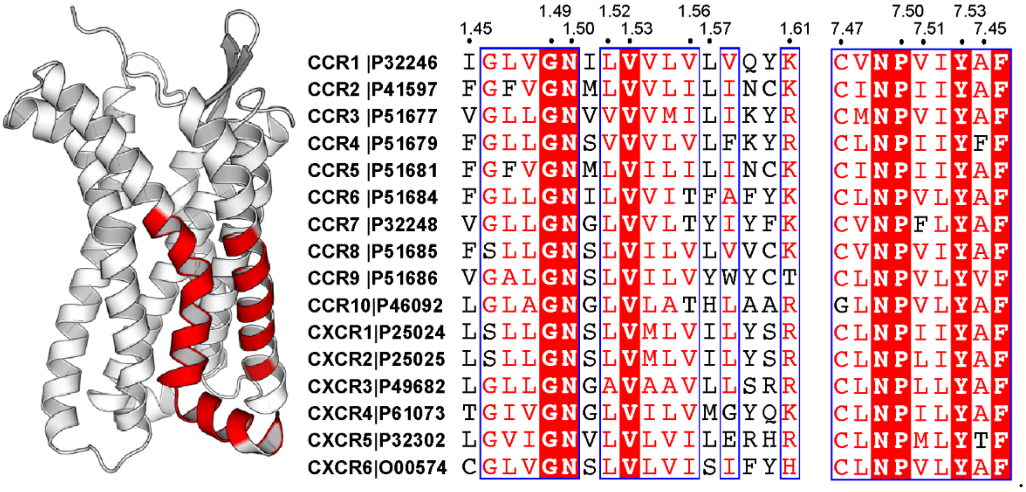

Figure S9. A Noncanonical Putative Cholesterol-Binding Site Previously Identified in CXCR4 and CCR5 [16] is accurately predicted by PyLipID analysis [8,9] of CCR3 and includes the amphipathic H8 (left). The main residues involved in this binding site (right) are largely conserved in CC and CXC motif chemokine receptors, number according to the Weinstein-Bellesteros numbering convention [15]. Listed are Uniprot accession numbers.

References

1. Williams, C.J.; Headd, J.J.; Moriarty, N.W.; Prisant, M.G.; Videau, L.L.; Deis, L.N.; Verma, V.; Keedy, D.A.; Hintze, B.; Chen, V.B.; et al. MolProbity: More and better reference data for improved all-atom structure validation. *Protein Sci.* **2017**, *27*, 293–315, doi:10.1002/pro.3330.
2. Tan, Q.; Zhu, Y.; Li, J.; Chen, Z.; Han, G.W.; Kufareva, I.; Li, T.; Ma, L.; Fenalti, G.; Zhang, W.; et al. Structure of the CCR5 Chemokine Receptor-HIV Entry Inhibitor Maraviroc Complex. *Sci.* **2013**, *341*, 1387–1390, doi:10.1126/science.1241475.
3. Trott, O.; Olson, A.J. AutoDock Vina: Improving the speed and accuracy of docking with a new scoring function, efficient optimization, and multithreading. *J. Comput. Chem.* **2009**, *31*, 455–461, doi:10.1002/jcc.21334.
4. Song, Y.; DiMaio, F.; Wang, R.Y.-R.; Kim, D.; Miles, C.; Brunette, T.; Thompson, J.; Baker, D. High-Resolution Comparative Modeling with RosettaCM. *Struct.* **2013**, *21*, 1735–1742, doi:10.1016/j.str.2013.08.005.
5. Wiederstein, M.; Sippl, M.J. ProSA-web: interactive web service for the recognition of errors in three-dimensional structures of proteins. *Nucleic Acids Res.* **2007**, *35*, W407–W410, doi:10.1093/nar/gkm290.
6. Laskowski, R.A.; Rullmann, J.A.C.; MacArthur, M.W.; Kaptein, R.; Thornton, J. AQUA and PROCHECK-NMR: Programs for checking the quality of protein structures solved by NMR. *J. Biomol. NMR* **1996**, *8*, 477–486, doi:10.1007/bf00228148.
7. Sterling, T.; Irwin, J.J. ZINC 15 – Ligand Discovery for Everyone. *J. Chem. Inf. Model.* **2015**, *55*, 2324–2337, doi:10.1021/acs.jcim.5b00559.
8. Ansell, T.B.; Song, W.; Sansom, M.S. The Glycosphingolipid GM3 Modulates Conformational Dynamics of the Glucagon Receptor. *Biophys. J.* **2020**, *119*, 300–313, doi:10.1016/j.bpj.2020.06.009.
9. Duncan, A.; Corey, R.A.; Sansom, M.S.P. Defining how multiple lipid species interact with inward rectifier potassium (Kir2) channels. *Proc. Natl. Acad. Sci.* **2020**, *117*, 7803–7813, doi:10.1073/pnas.1918387117.
10. Song, W.; Corey, R.A.; Duncan, A.L.; Ansell, T.B.; Stansfeld, P.J.; Sansom, M.S. Pylipid: A Python Toolkit for Analysis of Lipid-Protein Interactions from MD Simulations. *Biophys. J.* **2021**, *120*, 48a, doi:10.1016/j.bpj.2020.11.532.
11. Li, H.; Yao, Z.-X.; Degenhardt, B.; Teper, G.; Papadopoulos, V. Cholesterol binding at the cholesterol recognition/ interaction amino acid consensus (CRAC) of the peripheral-type benzodiazepine receptor and inhibition of steroidogenesis by an HIV TAT-CRAC peptide. *Proc. Natl. Acad. Sci.* **2001**, *98*, 1267–1272, doi:10.1073/pnas.031461598.
12. Fantini, J.; Epand, R.M.; Barrantes, F.J. Cholesterol-Recognition Motifs in Membrane Proteins. **2019**, *1135*, 3–25, doi:10.1007/978-3-030-14265-0_1.
13. Baier, C.J.; Fantini, J.; Barrantes, F.J. Disclosure of cholesterol recognition motifs in transmembrane domains of the human nicotinic acetylcholine receptor. *Sci. Rep.* **2011**, *1*, 69, doi:10.1038/srep00069.
14. Pándy-Szekeres, G.; Munk, C.; Tsonkov, T.; Mordalski, S.; Harpsøe, K.; Hauser, A.; Bojarski, A.; E Gloriam, D. GPCRdb in 2018: adding GPCR structure models and ligands. *Nucleic Acids Res.* **2017**, *46*, D440–D446, doi:10.1093/nar/gkx1109.
15. Ballesteros, J.A.; Weinstein, H. [19] Integrated methods for the construction of three-dimensional models and computational probing of structure-function relations in G protein-coupled receptors. **1995**, 366–428, doi:10.1016/s1043-9471(05)80049-7.

16. Zhukovsky, M.A.; Lee, P.-H.; Ott, A.; Helms, V. Putative cholesterol-binding sites in human immunodeficiency virus (HIV) coreceptors CXCR4 and CCR5. *Proteins: Struct. Funct. Bioinform.* **2012**, *81*, 555–567, doi:10.1002/prot.24211.
17. Zhukovsky, M.A.; Lee, P.H.; Ott, A.; Helms, V. Putative cholesterol-binding sites in human immunodeficiency virus (HIV) coreceptors CXCR4 and CCR5. *Proteins* **2013**, *81*, 555–567, doi:10.1002/prot.24211.
